# Supplementary material for: WHIM Syndrome-linked CXCR4 mutations drive osteoporosis
Source: Nat Commun. 2023 Apr 12;14:2058. doi: 10.1038/s41467-023-37791-4 (PMC10097661; doi:10.1038/s41467-023-37791-4)
Supplement: Supplementary file 1 — Supplementary Information [file 41467_2023_37791_MOESM1_ESM.pdf]

WHIM Syndrome-linked *CXCR4* mutations drive osteoporosis

SUPPLEMENTARY INFORMATION

Table S1: List of antibodies used for cell enrichment and flow cytometry.
Table S2: List of antibodies used for immunofluorescence.
Table S3: List of primers used for quantitative PCR.
Table S4: List of primers used for the BioMark assay.
Figure S1: Cxcr4-mediated signaling is dysregulated in *Cxcr4*^{1013}-bearing skeletal cells.
Figure S2: *In vitro* functional capacities of *Cxcr4*^{1013}-bearing osteoclasts and osteoprogenitor cells.
Figure S3: Transcriptional signatures in *Cxcr4*^{1013}-bearing skeletal stromal/stem cells.
Figure S4: *In vitro* differentiation capacities of *Cxcr4*^{1013}-bearing skeletal stromal/stem cells.
Figure S5: *In vitro* characterization of bone marrow stromal cells from patients with WHIM Syndrome.

SUPPLEMENTARY TABLES

<u>Table S1:</u> List of antibodies used for cell enrichment and flow cytometry.

Anti-mouse Ab	clone	Isotype	Supplier	Catalog #	Dilution
CD11b	M1/70	rat IgG2b	eBioscience	13-0112-82	1:500
CD31	MEC 13.3	rat IgG2a	BD Pharmingen	553372	1:200
CD34	RAM34	rat IgG2a	Invitrogen	11-0341-82	1:200
CD45	30F11	rat IgG2b	Sony	1115640	1:400
CD45.1	A20	mouse IgG2a	Sony	1153620	1:300
CD45.2	104	mouse IgG2a	Biolegend	109829	1:300
CD48	HM48-1	Armenian hamster IgG	BD Pharmingen	747718	1:200
CD51	RMV-7	rat IgG1	Sony	1120530	1:100
CD71	C2	rat IgG1	BD Pharmingen	562858	1:200
CD117	2B8	rat IgG2b	Sony	1129120	1:50
CD135	A2F10	rat IgG2a	Biolegend	135305	1:50
CD140a	APA5	rat IgG2a	BD Horizon	558774	1:200
CD150	TC15-23F12,2	rat IgG2a	Sony	1179515	1:100
Cxcr4	2B11	rat IgG2b	BD Biosciences	551966	1:100
Ackr3	8F11-M16	mouse IgG2b	BioLegend	331103	1:200
KI67	B56	mouse IgG1	BD Biosciences	563756	1:200
Sca-1	E13-161.7	Rat IgG2a	Sony	1212570	1:400
Ter119	TER-119	rat IgG2b	Sony	1181140	1:400
Phospho-Erk	20A	mouse IgG1	BD Biosciences	612566	1:25
**Anti-human Ab**	**clone**	**Isotype**	**Supplier**	**Catalog #**	**Dilution**
CD45	HI30	mouse IgG1	BD Biosciences	563879	1:25
CD73	AD2	mouse IgG1	BD Biosciences	561254	1:25
CD90	5E10	mouse IgG1	BioLegend	328117	1:25
CD105	266	mouse IgG1	BD Biosciences	563466	1:25
CXCR4	12G5	mouse IgG2a	BD Biosciences	555976	1:25
ACKR3/CXCR7	8F11-M16	mouse IgG2b	BioLegend	331104	1:25

Anti-mouse Lin cocktail: anti-CD3, anti-CD45R, anti-CD11b, anti-TER119, anti-CD41 and anti-Gr-1 mAbs (BD Biosciences)

<u>Table S2:</u> List of antibodies used for immunofluorescence.

Origin	Antibody	Conjugate	Supplier	Catalog #	Dilution
Mouse	Cxcl12	Purified	R&D	MAB350	1:30
pGoat	Opn	Purified	R&D	AF808-SP	1:50
pGuinea pig	Perilipin A	Purified	Research Diagnostic Inc	RDIPROGP29	1:5000
pRabbit	Osterix	purified	Santa Cruz	SC-22536R	1:200
Mouse	IgG1	Purified	Invitrogen	MA1-34581	1:200
pGoat	Mouse	AF633	Invitrogen	A-21235	1:200
pRabbit	Guinea pig	TRITC	OriGene Technologies	R1322T	1:5000
pDonkey	rabbit	Dylight 550	Thermofisher	SA5-10039	1:1000

Table S3: **List of primers used for quantitative PCR.**

Genes	Forward 5'->3'	Reverse 5'->3'
**Mouse (m) and human (h) osteogenic differentiation**		
mOcn	GGGCAATAAGGTAGTGAACAG	GCAGCACAGGTCCTAAATAGT
hOCN	CACCGAGACACCATGAGAGC	CTGGGTCTCTTCACTACCTC
mAlp	CACAATATCAAGGATATCGACGTGA	ACATCAGTTCTGTTCTTCGGGTACA
mOsx	ATGGCGTCCTCTCTGCTTGA	GAAGGGTGGGTAGTCATTTG
hOSX	TGCTTGAGGAGGAAGTTCAC	AGGTCACTGCCCACAGAGTA
mOpn	GCCTGTTTGGCATTGCCTCCTC	CACAGCATTCTGTGGCGCAAGG
hOPN	TCTAAGAAGTTTCGCAGACC	ATGTCCTCGTCTGTAGCATC
mRunx2	ACGAGGCAAGAGTTTCACC	GGACCGTCCACTGTCACTTT
hRUNX2	AGTGGACGAGGCAAGAGTTTCA	GGGTTCCCGAGGTCCATCTA
**Mouse (m) chondrogenic differentiation**		
mSox9	TACGACTGGACGCTGGTGCC	CCGTTCTTCACCGACTTCCTCC
mAggrecan	GCCTCTCAAGCCCTTGTCTG	CACCCCTCCTCACATTGCTC
mCol2α1	CTGACCTGACCTGATGATACC	CACCAGATAGTTCCTGTCTCC
**Mouse (m) adipogenic differentiation**		
mPparg	GACCACTCGCATTCCTTT	CCACAGACTCGGCACTCA
mFabp4	CTTGTGGAAGTCACGCCTTT	AAGAGAAAACGAGATGGTGACAA
mPln1	AGCGTGGAGAGTAAGGATGTC	CTTCTGGAAGCACTCACAGG
**Mouse (m) MSC markers**		
mCd51	ACCACTAACATCACCTGGGG	TCTTCTTGAGGTGGTCGGAC
mSca-1	GCTGATTCTTCTTGTGGCCC	CCACAATAACTGCTGCCTCC
mPdgfrα	CGACTGGATGATCTGCAAGC	GCTGAGGTTGTTCTTTGCCA
**Mouse (m) osteoclastogenic differentiation**		
mTnfsf11	GGAACTGCAACACATTGTGGG	GCCTTCCATCATAGCTGGAGC
mTnfrs11b	TCCGGCGTGGTGCAA	AGAACCCATCTGGACATTTTTTG
mCsf1	TTAAAGACAACACCCCCAATGC	TCAGGTTATTGGAGAGTTCCTGGA
**Mouse (m) osteoclastic differentiation**		
mNfatc1	TGAGGCTGGTCTTCCGAGTT	CGCTGGGAACACTCGATAGG
mClcn7	CTTGAAGCATAAGGTGTTTGTGGA	CTCAGTCGCCGCTGCAC
mCtsk	CAGCAGAGGTGTGTACTATG	GCGTTGTTCTTATTCCGAGC
mTnfrsf11a	CTTGGACACCTGGAATGAAGAAG	AGGGCCTTGCCTGCATC
**Mouse (m) and human (h) housekeeping genes**		
mActin	GGGTCAGAAGGACTCCTATG	GGTCTCAAACATGATCTGGG
hACTIN	AGTCATTCCAAATATGAGATGCGTT	TGCTATCACCTCCCCTGTGT
m36b4	TCCAGGCTTTGGGCATCA	CTTTATCAGCTGCACATCACTCAGA
hGAPDH	TGCACCACCAACTGCTTAGC	GGCATGGACTGTGGTCATGAG

<u>Table S4:</u> List of primers used for the BioMark assay.

Genes	References	Supplier
**Osteogenic markers**		
Col1 α	Mm00801666_g1	Applied Biosystems
Alp	Mm00475834_m1	Applied Biosystems
Ibsp	Mm00492555_m1	Applied Biosystems
Runx2	Mm00501584_m1	Applied Biosystems
Dmp1	Mm01208363_m1	Applied Biosystems
**Cell cycle**		
Ccnd2	Mm00438070_m1	Applied Biosystems
Ccnd3	Mm01612362_m1	Applied Biosystems
**Irrelevant genes (negative controls)**		
Pax5	Mm00435501_m1	Applied Biosystems
CD3e	Mm00599684_g1	Applied Biosystems
**Housekeeping gene**		
Actin β	Mm01205647_g1	Applied Biosystems

LEGENDS TO SUPPLEMENTARY FIGURES

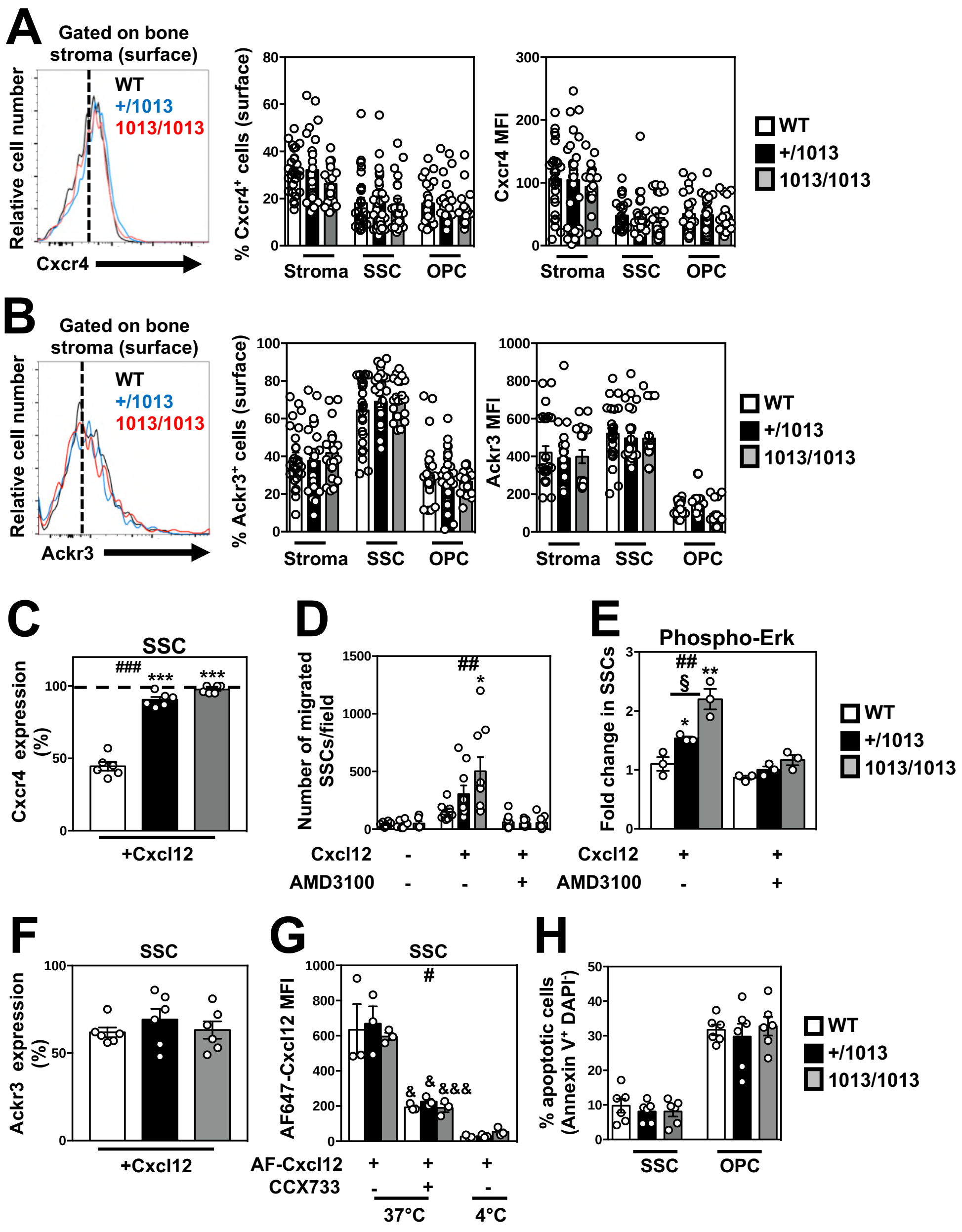

Figure S1: Cxcr4-mediated signaling is dysregulated in *Cxcr4*^{1013}-bearing skeletal cells. (A and B) Expression levels of Cxcr4 (A) or Ackr3 (B) were determined by flow cytometry on gated (Ter119^{-}CD45^{-}) stromal cells, skeletal stromal/stem cells (SSCs) and osteoblast progenitor cells (OPCs) from bone fractions of WT and mutant mice. Left: Representative histograms for surface detection of Cxcr4 or Ackr3 on gated bone stromal cells. Background fluorescence is shown (isotype, dotted vertical line). Middle and right: Cxcr4- or Ackr3-positive fractions or mean fluorescence intensity (MFI) values obtained within bone stromal cells, SSCs and OPCs relative to background fluorescence based on the corresponding isotype control staining. Data (means $\pm$ SEM) are from at least ten independent experiments with n= 27, 28, and 21 mice in total for WT, +/1013 and 1013/1013 groups, respectively. **(C)** Cell surface expression of Cxcr4 on SSCs upon exposure to 10 nM Cxcl12 at 37°C for 45 min. Cxcr4 expression on bone cells incubated in medium alone was set at 100% (dotted horizontal line). Data (means $\pm$ SEM) are pooled from three independent experiments with six mice in total per group. Statistics were calculated with the nonparametric Kruskal–Wallis H test ($^{###}$p<0.0001)

and the unpaired two-tailed Student's t test (+/1013 vs WT and 1013/1013 vs WT ***p<0.0001). **(D)** Migration of cultured WT or mutant SSCs in response to 1 nM Cxcl12 in the presence or absence of 10 µM AMD3100 was assessed in two or three independent fields after crystal violet staining. Data (means ± SEM) are from three independent SSC cultures per genotype. Statistics were calculated with the nonparametric Kruskal–Wallis H test ($^{\#\#}$p=0.0092) and the unpaired two-tailed Student's t test (1013/1013 vs WT *p=0.0124). **(E)** *In vitro* expanded SSCs from bone fractions of WT or mutant mice pre-incubated or not with 10 µM AMD3100 were stimulated 2 min with 10 nM Cxcl12 at 37°C and then the MFI values of phospho-Erk were determined by flow cytometry and represented as a fold change expression. Data (means ± SEM) are from three independent SSC cultures per genotype. Statistics were calculated with the nonparametric Kruskal–Wallis H test ($^{\#\#}$p=0.0036) and the unpaired two-tailed Student's t test (+/1013 vs WT *p=0.022, 1013/1013 vs WT **p=0.0062, +/1013 vs 1013/1013 §p=0.0194). **(F)** Cell surface expression of Ackr3 on SSCs upon exposure to 10 nM Cxcl12 at 37°C for 45 min. Ackr3 expression on bone cells incubated in medium alone was set at 100% (dotted horizontal line). Data (means ± SEM) are from three independent experiments with six mice in total per group. **(G)** Cultured WT or mutant SSCs were pre-treated or not with 100 µM of the Ackr3 antagonist CCX733 and then incubated with 5 nM Cxcl12-AF647 at 37°C for 60 min. Cells were washed with an acidic glycine buffer to remove cell surface-bound Cxcl12-AF647. Geometric MFI values for Cxcl12-AF647 were determined by flow cytometry. No Cxcl12-AF647 uptake was observed in SSCs incubated at 4°C. Data (means ± SEM) are pooled from three individual SSC cultures per genotype. Statistics were calculated with the nonparametric Kruskal–Wallis H test ($^{\#}$p=0.018) and the unpaired two-tailed Student's t test (WT treated vs WT untreated $^{\&}$p=0.039, +/1013 treated vs +/1013 untreated $^{\&}$p=0.0113, 1013/1013 treated vs 1013/1013 untreated $^{\&\&\&}$p=0.0002). **(H)** Flow-cytometric determination of the proportions of apoptotic (Annexin V^{+} DAPI^{-}) SSCs and OPCs from bone fractions of WT and mutant mice. Data (means ± SEM) are from two independent experiments with six mice in total per group. Mice were littermates, females and age-matched (8-12 wk-old). Source data are provided as a Source Data file.

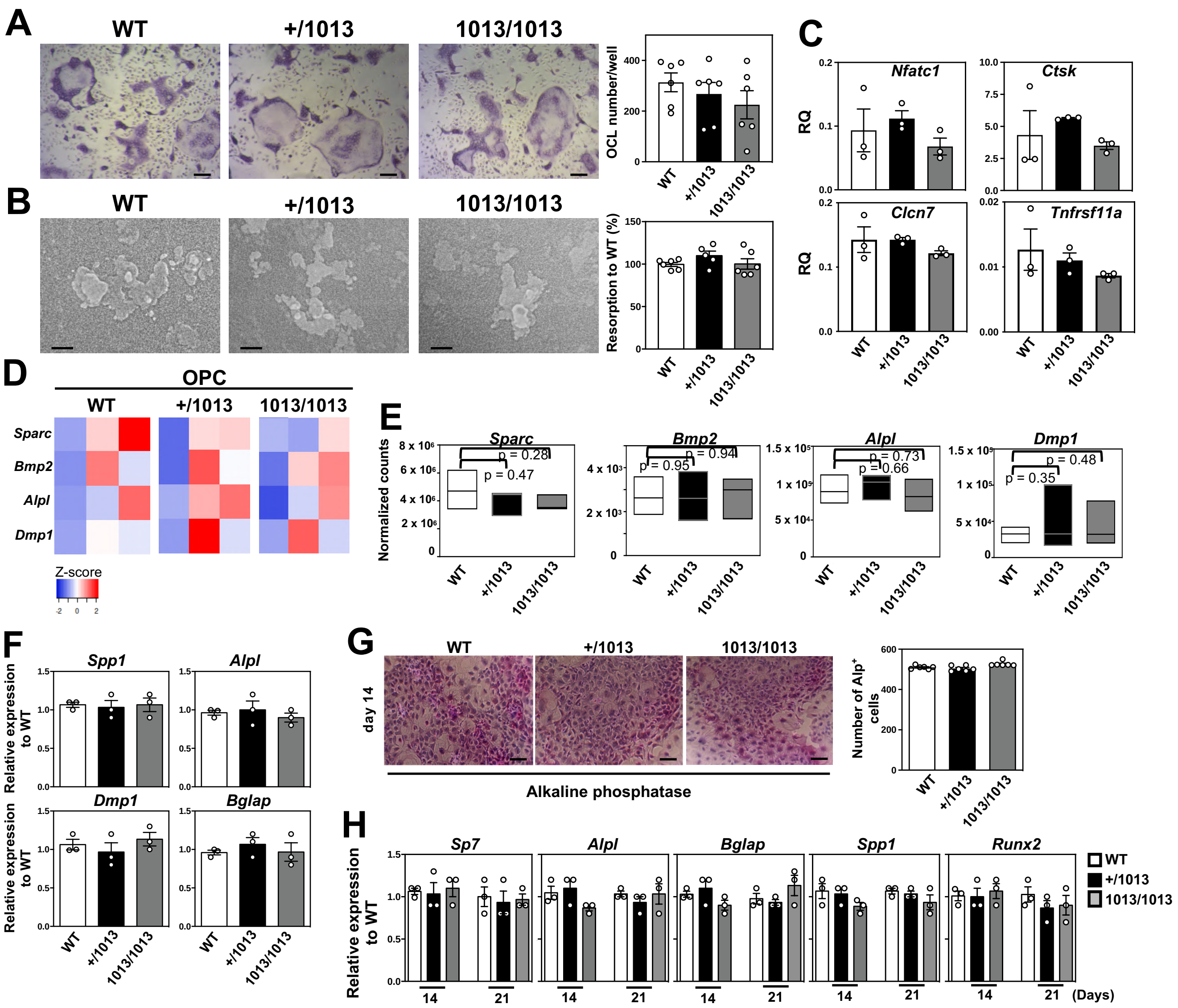

Figure S2: *In vitro* functional capacities of *Cxcr4*^{1013}-bearing osteoclasts and osteoprogenitor cells. (A) Total BM cells were differentiated for 5 days in osteoclastic medium and OCLs (TRAP-positive) were identified (left, representative images, bars: 100 µm) and quantified (right). Data (means ± SEM) are from 2 independent experiments with 6 mice in total per group. **(B)** *In vitro* differentiated OCLs were analyzed for their resorptive capacity of a mineralized matrix. Pictures show the resorptive lacunae produced by OCLs (left, representative images, bars: 100 µm). The proportion of lacunae surface relative to the whole surface was calculated and expressed as a percentage of the mineral area resorbed by WT OCLs (right panel). Data (means ± SEM) are from 2 independent experiments with n= 6, 5, and 6 mice in total for WT, +/1013 and 1013/1013 groups, respectively. **(C)** Relative expression levels (RQ) of osteoclastic genes were determined in osteoclastic differentiation cultures (3 mice per group) by quantitative PCR. Each individual sample was run in triplicate and has been standardized for *36B4* expression levels. **(D)** RNA-seq-based heatmap representing the relative expression levels of mineralization genes expressed by sorted OPCs performed on three biological replicates per group with one replicate representing the pool of 3 mice. **(E)** Normalized counts of selected mineralization genes using the DESeq2 method. Data are represented as floating bars (min to max and line equal median) of the 3 biological replicates per group. For significance testing, DESeq2 uses a Wald test (p values). The Wald test P values

from the subset of genes that pass an independent filtering step, are adjusted for multiple testing using the procedure of Benjamini and Hochberg (padj values). **(F)** Expression levels of osteogenic genes were determined by quantitative PCR (3 mice per group). Each individual sample was run in triplicate and was standardized for β-actin expression levels. Results (means ± SEM) are expressed as relative expression compared to WT samples. **(G)** Alkaline phosphatase (Alp) staining was performed 14 days after initiation of the culture of WT and mutant OPCs in osteogenic medium (bars: 100 μm). Quantitative analyses (number of Alp+ cells) were performed under an inverted microscope. Data (means ± SEM) are from 6 independent cultures per genotype. **(H)** Expression levels of osteogenic and mineralization genes were determined by quantitative PCR in OPCs 14 and 21 days after initiation of the osteogenic culture (3 independent cultures per group). Each individual sample was run in triplicate and was standardized for β-actin expression levels. Results (means ± SEM) are expressed as relative expression compared to WT samples. Mice were littermates, females and age-matched (8-12 wk-old). Source data are provided as a Source Data file.

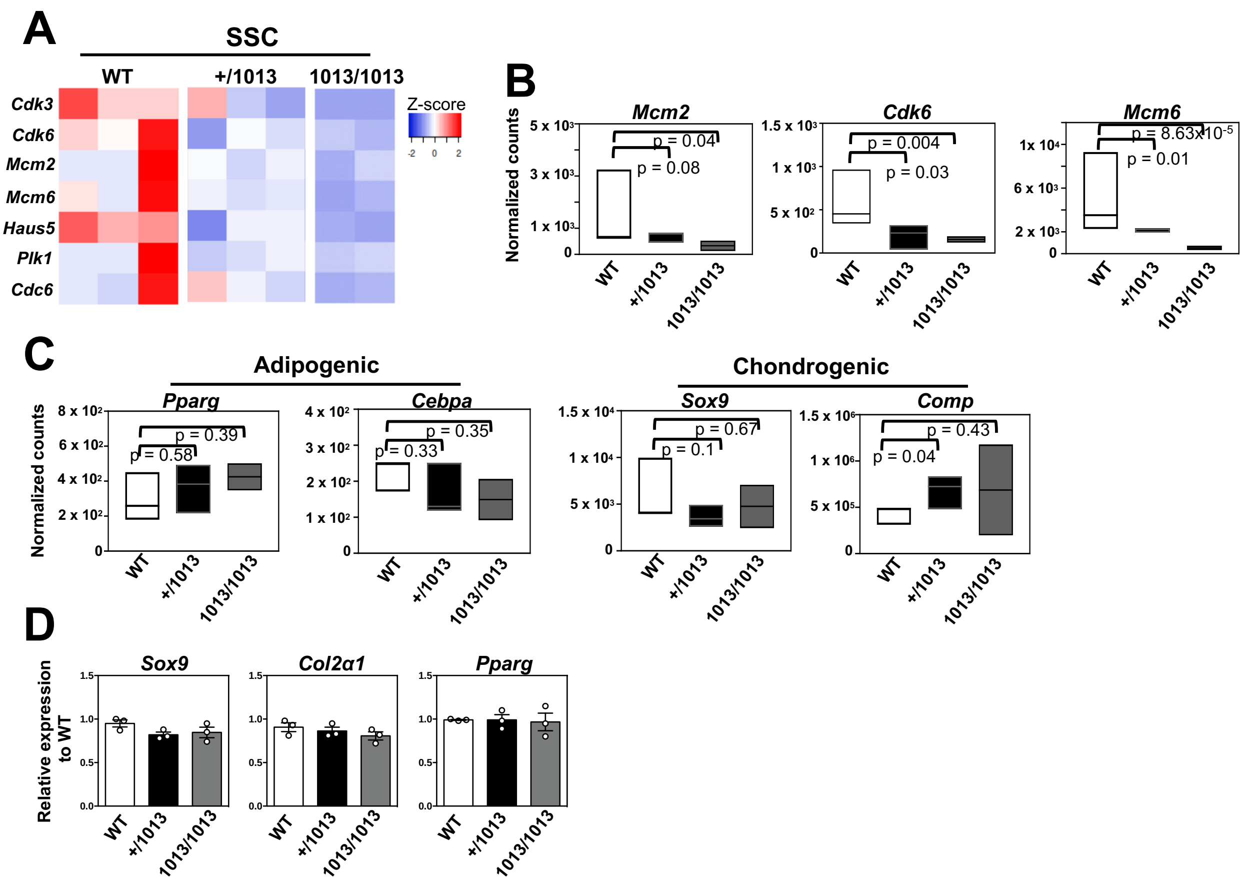

Figure S3: Transcriptional signatures in *Cxcr4*^{1013}-bearing skeletal stromal/stem cells. (A) RNA-seq-based heatmap representing the relative expression levels of cell cycle genes expressed by sorted SSCs from WT and mutant mice. Two or three biological replicates per group have been performed with one replicate representing the pool of 3 mice. **(B)** Normalized counts of selected cell cycle genes using the DESeq2 method. **(C)** Normalized counts of selected adipogenic and chondrogenic differentiation genes using the DESeq2 method. Data in B and C are represented as floating bars (min to max and line equal median) of the 2 or 3 biological replicates per group. For significance testing, DESeq2 uses a Wald test (p values). The Wald test P values from the subset of genes that pass an independent filtering step, are adjusted for multiple testing using the procedure of Benjamini and Hochberg (padj values). **(D)** Relative expression of selected adipogenic and chondrogenic genes in WT and mutant SSCs obtained by quantitative PCR (3 mice per group). Each individual sample was run in triplicate and has been standardized for β-actin expression levels. Results (means $\pm$ SEM) are expressed as relative expression compared to WT samples. Mice were littermates, females and age-matched (8-12 wk-old). Source data are provided as a Source Data file.

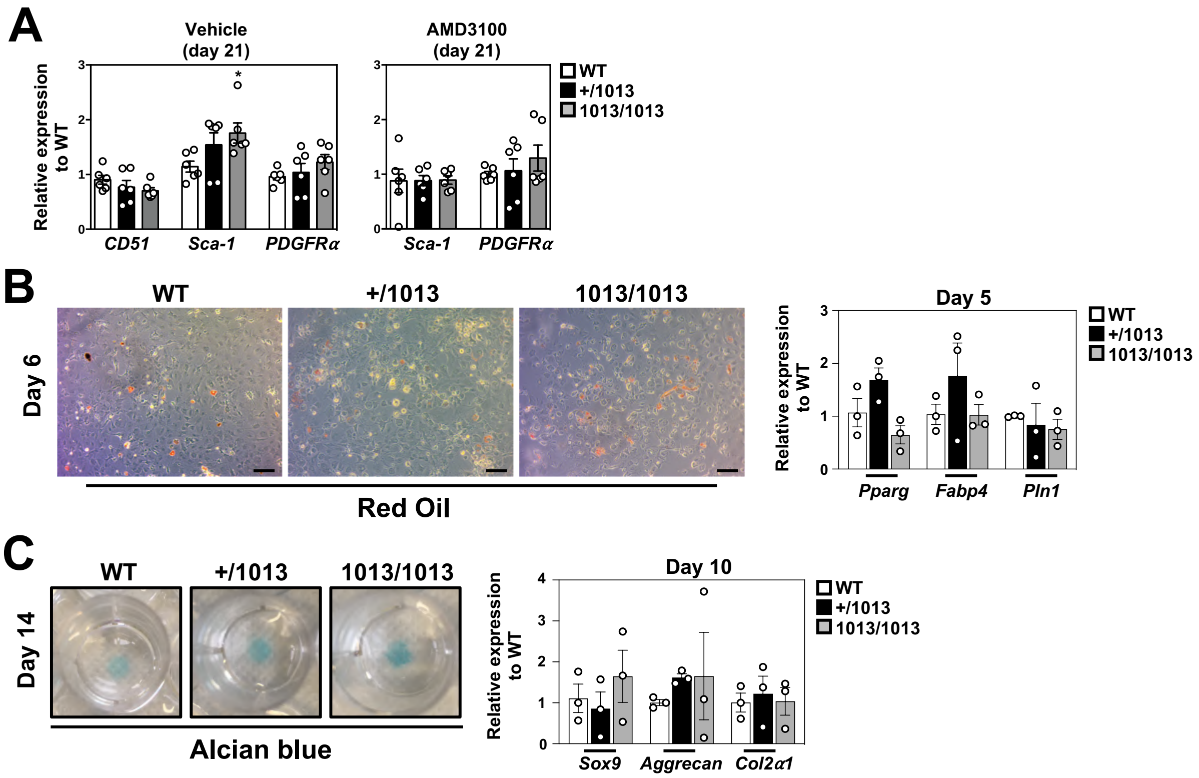

Figure S4: ***In vitro*** **differentiation capacities of** ***Cxcr4*^{1013}-bearing skeletal stromal/stem cells.** **(A)** Expression levels of stromal genes (*CD51, Sca-1* and *PDGFRα*) were determined by quantitative PCR in 6 independent SSC cultures per genotype after 21 days of osteogenic culture in the presence or absence of AMD3100. Each individual sample was run in triplicate and was standardized for *β-actin* expression levels. Results (means ± SEM) are expressed as relative expression to WT samples. Statistics were calculated with the unpaired two-tailed Student's t test (1013/1013 vs WT *p=0.0149). **(B)** Left: Oil Red O staining was performed 6 days after initiation of cultures of WT and mutant SSCs in adipogenic medium (bars: 100 µm). Right: Expression levels of adipogenic genes (*Pparg, Fabp4* and *Pln1*) were determined by quantitative PCR in WT and mutant cultures at day 5. Each individual sample was run in triplicate and has been standardized for *36b4* expression levels. Results (means ± SEM) are expressed as relative expression to WT samples and are from 3 independent SSC cultures per genotype. **(C)** Left: Alcian blue staining was performed 14 days after initiation of the culture of WT and mutant SSCs in chondrogenic medium. Right: Expression levels of chondrogenic genes (*Sox9*, *Aggrecan* and *Col2α1*) were determined by quantitative PCR in WT and mutant cultures at day 10. Each individual sample was run in triplicate and has been standardized for *36b4* expression levels. Results (means ± SEM) are expressed as relative expression to WT samples and are from 3 independent SSC cultures per genotype. Mice were littermates, females and age-matched (8-12 wk-old). Source data are provided as a Source Data file.

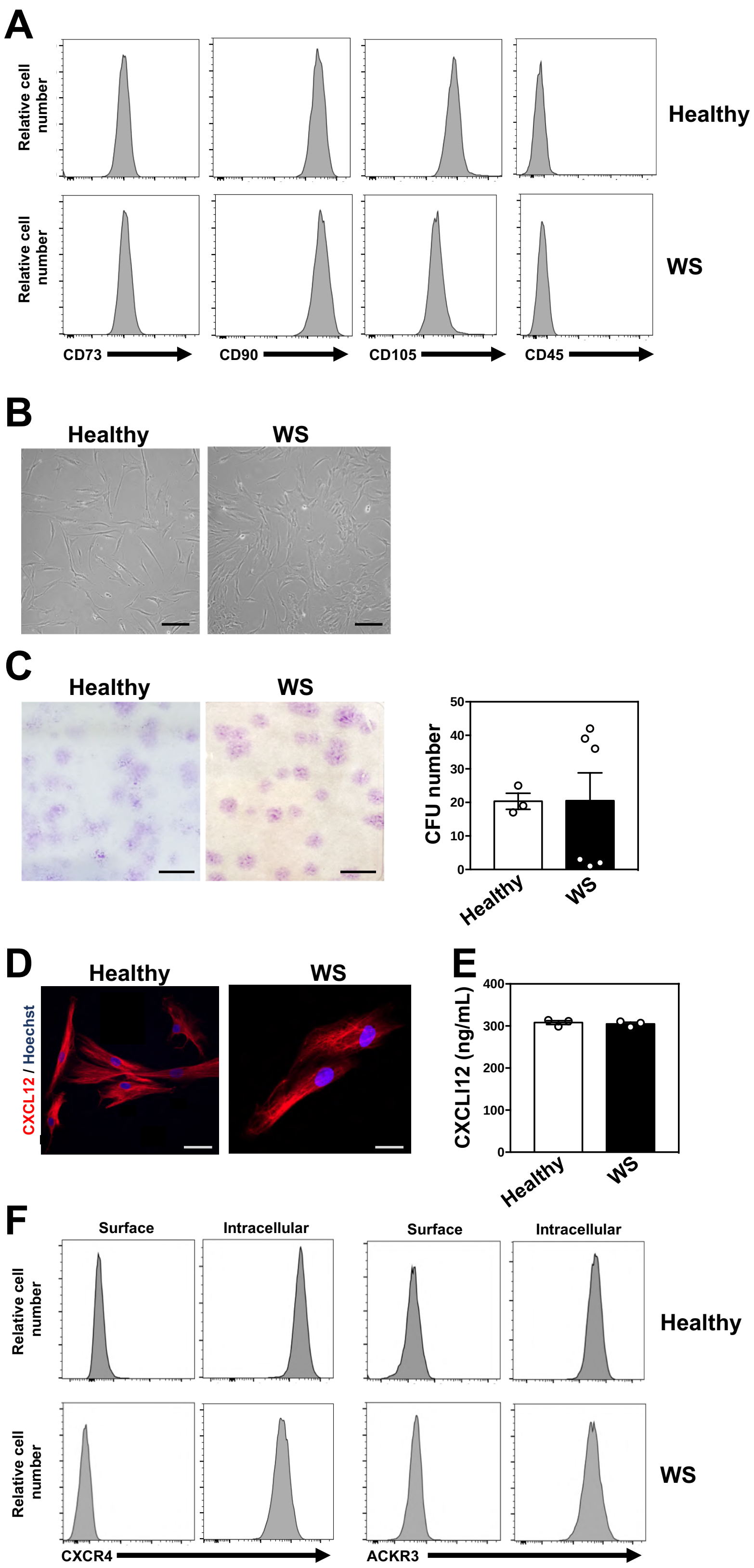

Figure S5: ***In vitro* characterization of bone marrow stromal cells from patients with WHIM Syndrome. (A)** Surface detection of CD73, CD90 and CD105 was determined by flow cytometry on *in vitro* expanded bone marrow stromal cells (BMSCs) from a representative healthy donor (top) or WHIM Syndrome (WS) patient (bottom). BMSCs were negative for the hematopoietic marker CD45. **(B)** Bright field pictures of the corresponding primary healthy and

WS BMSC cultures. Healthy and WS BMSCs were all spindle shaped and fibroblast-like cells. At all culture passages, WS MSCs exhibited a fibroblast-like morphology. Bars: 200 µm. Pictures are representative of 7 healthy and 2 WS BMSC cultures. **(C)** Representative crystal violet staining of colonies formed from 0.2 x 10^3 BMSCs of healthy or WS donors (left). Bars: 1 cm. Quantification of the number of colonies (means $\pm$ SEM) obtained in these CFU-F assays (right) from one healthy and two WS donors. Each individual sample has been run in triplicate. **(D and E)** CXCL12 expression were determined in BMSC cultures by immunofluorescence and ELISA. Immunofluorescence staining of CXCL12 in association with Hoechst 33342 in healthy and WS BMSC cultures is shown (D, bars: 50 µm). Images are representative of five independent determinations. CXCL12 secreted in the supernatants from BMSC cultures of healthy and WS donors were determined by ELISA (E). Data (means $\pm$ SEM) are from two independent cultures. **(F)** Expression levels of CXCR4 (left) or ACKR3 (right) were determined by flow cytometry in BMSCs from healthy and WS donors before (surface) and after (intracellular) permeabilization. Representative histograms for surface or intracellular detection of CXCR4 or ACKR3 are shown. Source data are provided as a Source Data file.
