# Supplementary material for: WHIM Syndrome-linked CXCR4 mutations drive osteoporosis
Source: Nat Commun. 2023 Apr 12;14:2058. doi: 10.1038/s41467-023-37791-4 (PMC10097661; doi:10.1038/s41467-023-37791-4)
Supplement: Supplementary file 3 — Reporting Summary [file 41467_2023_37791_MOESM3_ESM.pdf]

nature portfolio

Corresponding author(s): Karl BALABANIAN

Last updated by author(s): Feb 28, 2023

Reporting Summary

Nature Portfolio wishes to improve the reproducibility of the work that we publish. This form provides structure for consistency and transparency in reporting. For further information on Nature Portfolio policies, see our Editorial Policies and the Editorial Policy Checklist.

Statistics

For all statistical analyses, confirm that the following items are present in the figure legend, table legend, main text, or Methods section.

n/a	Confirmed	
☐	☒	The exact sample size (*n*) for each experimental group/condition, given as a discrete number and unit of measurement
☐	☒	A statement on whether measurements were taken from distinct samples or whether the same sample was measured repeatedly
☐	☒	The statistical test(s) used AND whether they are one- or two-sided<br>*Only common tests should be described solely by name; describe more complex techniques in the Methods section.*
☐	☒	A description of all covariates tested
☐	☒	A description of any assumptions or corrections, such as tests of normality and adjustment for multiple comparisons
☐	☒	A full description of the statistical parameters including central tendency (e.g. means) or other basic estimates (e.g. regression coefficient) AND variation (e.g. standard deviation) or associated estimates of uncertainty (e.g. confidence intervals)
☐	☒	For null hypothesis testing, the test statistic (e.g. *F*, *t*, *r*) with confidence intervals, effect sizes, degrees of freedom and *P* value noted<br>*Give P values as exact values whenever suitable.*
☒	☐	For Bayesian analysis, information on the choice of priors and Markov chain Monte Carlo settings
☒	☐	For hierarchical and complex designs, identification of the appropriate level for tests and full reporting of outcomes
☒	☐	Estimates of effect sizes (e.g. Cohen's *d*, Pearson's *r*), indicating how they were calculated

Our web collection on statistics for biologists contains articles on many of the points above.

Software and code

Policy information about availability of computer code

Data collection	FACS Diva software version 7 (BD), ICY and Bonolab softwares
Data analysis	FLOWJO v10.7 (BD), GraphPad Prism v8.0e (GraphPad software Inc.), Fiji, ImageJ, Leica Application Suite V3.8, and Htseq softwares

For manuscripts utilizing custom algorithms or software that are central to the research but not yet described in published literature, software must be made available to editors and reviewers. We strongly encourage code deposition in a community repository (e.g. GitHub). See the Nature Portfolio guidelines for submitting code & software for further information.

Data

Policy information about availability of data

All manuscripts must include a data availability statement. This statement should provide the following information, where applicable:

- Accession codes, unique identifiers, or web links for publicly available datasets
- A description of any restrictions on data availability
- For clinical datasets or third party data, please ensure that the statement adheres to our policy

RNA-seq data that support the findings of this study have been deposited in the Gene Expression Omnibus repository with the accession code GSE217422 (https://www.ncbi.nlm.nih.gov/geo/query/acc.cgi?acc=GSE217422). Source data are provided with this paper.

Human research participants

Policy information about studies involving human research participants and Sex and Gender in Research.

Reporting on sex and gender	For BMD assessment, data were collected from 13 women and 6 men with an average age of 30.5 years (range 10-56). There was no bias of recruitment in terms of sex or gender.
Population characteristics	Population characteristics relevant for this study were age, sex (biological attribute), diagnosis of WHIM Syndrome, CXCR4 mutation pattern and treatments.
Recruitment	The WHIM diagnosis is the sole criteria of selection and inclusion.
Ethics oversight	The study was approved by the NIAID Institutional Review Board and the Ethical Board Ile-de-France X. The study was conducted in accordance with Helsinki's declaration. Recruited WHIM Syndrome patients were not compensated and gave their written informed consent for participating to the clinical study and samples were anonymized for the biological analyses.

Note that full information on the approval of the study protocol must also be provided in the manuscript.

Field-specific reporting

Please select the one below that is the best fit for your research. If you are not sure, read the appropriate sections before making your selection.

☒ Life sciences ☐ Behavioural & social sciences ☐ Ecological, evolutionary & environmental sciences

For a reference copy of the document with all sections, see nature.com/documents/nr-reporting-summary-flat.pdf

Life sciences study design

All studies must disclose on these points even when the disclosure is negative.

Sample size	Cryopreserved BM aspirates from two WS patient (NIH protocol 09-I-0200) were provided by Drs. D.H. McDermott and P.M. Murphy through a NIH Material Transfer Agreement. BM samples from seven healthy donors that were matched for age and sex and used as control subjects were isolated from hip replacement surgery samples (Protocol 17-030, n° ID-RCB: 2017-A01019-44). Primary BMSCs from healthy and WS donors were amplified and used at passage 1 to 3. For BMD assessment, data were collected from nineteen WS patients as part of an IRB approved clinical protocol conducted at the NIH (NIAID Protocol #2014-I-0185, IND # 118767). Patients had a baseline bone density scan as part of a drug treatment trial (NCT02231879) comparing 1 year of twice daily filgrastim (Neupogen) versus plerixafor (Mozobil) in a randomized, blinded crossover design. There were 13 women and 6 men with an average age of 30.5 years (range 10-56). Patients had been on filgrastim (Neupogen) for an average of 5.7 years prior to enrolling in the trial (range 0-27). 6 of the 19 had not used filgrastim regularly prior to trial enrollment. BMD values expressed as T- or Z-scores were measured by total body dual-energy X-ray absorptiometry with a Lunar iDXA densitometer (GE Healthcare). Five WS patients had abnormal screening bone density by WHO criteria, anonymized at the start of the Phase 3 trial (Table 1), while the other 14 patients had normal bone density (not shown). No statistical methods were used to determine sample sizes. Sample sizes were determined based on the number of samples available as well as on the number of patients recruited in the clinical trial. Note that the WHIM Syndrome is an extremely rare immune disorder with only <200 cases reported in the litterature.
Data exclusions	No data were excluded in this study
Replication	BMD measurements by total body dual-energy X-ray absorptiometry of WS patients has been carried out once. For in vitro assays, we were limited the rarity of the disorder as well as to the origin (ie., bone marrow) of the sample. That's why we investigated cryopreserved BM aspirates from two WS patient. However, all attempts at replication were successful in BMSC derived from these samples.
Randomization	Randomization was not possible in this study. WHIM Syndrome is an ultrarare disease. As a consequence, we had only two suitable bone marrow samples from WHIM patients for study. These had previously been collected and were cryopreserved at the NIH, then sent to France for analysis. They were designated as the WHIM samples and were compared to the age and sex matched healthy controls collected in France.
Blinding	Blinding to group allocation is not relevant to this study since the samples were already obtained from the patients and there was no intervention of the patients to which the investigators could be blinded. The WHIM samples were anonymized to the French investigators on the study. They were identified only as WHIM samples and blinded to other potential identifiers.

Reporting for specific materials, systems and methods

We require information from authors about some types of materials, experimental systems and methods used in many studies. Here, indicate whether each material, system or method listed is relevant to your study. If you are not sure if a list item applies to your research, read the appropriate section before selecting a response.

Materials & experimental systems

n/a	Involved in the study
☐	☒ Antibodies
☒	☐ Eukaryotic cell lines
☒	☐ Palaeontology and archaeology
☐	☒ Animals and other organisms
☒	☐ Clinical data
☒	☐ Dual use research of concern

Methods

n/a	Involved in the study
☒	☐ ChIP-seq
☐	☒ Flow cytometry
☒	☐ MRI-based neuroimaging

Antibodies

Antibodies used

Anti-mouse Antibody / clone / Isotype / Supplier / catalog # / dilution (used in FCM and cell enrichment experiments)
CD11b/ M1/70 /rat IgG2b /eBioscience /13-0112-82/ 1:500
CD31 /MEC 13.3/ rat IgG2a /BD Pharmingen /553372/ 1:200
CD34/ RAM34/ rat IgG2a /Invitrogen /11-0341-82 /1:200
CD45 /30F11/ rat IgG2b /Sony /1115640 /1:400
CD45.1/ A20/ mouse IgG2a /Sony /1153620 /1:300
CD45.2 /104/ mouse IgG2a /Biolegend /109829 /1:300
CD48 /HM48-1/ Armenian hamster IgG /BD Pharmingen /747718 /1:200
CD51 / RMV-7 /rat IgG1 /Sony /1120530 /1:100
CD71 /C2 /rat IgG1 /BD Pharmingen/ 562858 /1:200
CD117 /2B8 /rat IgG2b /Sony /1129120 /1:50
CD135/ A2F10 /rat IgG2a /Biolegend/ 135305 /1:50
CD140a/ APA5 /rat IgG2a /BD Horizon/ 558774 /1:200
CD150 /TC15-23F12,2 /rat IgG2a /Sony /1179515 /1:100
Cxcr4/ 2B11 /rat IgG2b /BD Biosciences/ 551966 /1:100
Ackr3/ 8F11-M16/ mouse IgG2b /BioLegend/ 331103 /1:200
KI67/ B56/ mouse IgG1 /BD Biosciences/ 563756 /1:200
Sca-1/ E13-161.7 /Rat IgG2a /Sony /1212570 /1:400
Ter119 /TER-119 /rat IgG2b/ Sony /1181140 /1:400
Phospho-Erk/ 20A /mouse IgG1 /BD Biosciences /612566 /1:25

Anti-human Antibody / clone / Isotype / Supplier/ catalog # / dilution (used in FCM experiments)
CD45 /HI30/mouse IgG1/ BD Biosciences/ 563879/1:25
CD73 /AD2/ mouse IgG1/ BD Biosciences/ 561254/1:25
CD90 /5E10/ mouse IgG1/ BioLegend /328117/ 1:25
CD105/ 266/ mouse IgG1/ BD Biosciences/ 563466/1:25
CXCR4 /12G5/ mouse IgG2a/ BD Biosciences/ 555976/ 1:25
ACKR3-CXCR7 /8F11-M16/ mouse IgG2b / BioLegend /331104/ 1:25

Origin/Antibody Conjugate/ Supplier/ Catalog # /Dilution (used for immunofluorescence studies)
Mouse/ Cxcl12/ Purified/ R&D /MAB350 /1:30
pGoat/ Opn/ Purified /R&D /AF808-SP/ 1:50
pGuinea pig /Perilipin A/ Purified /Research Diagnostic Inc/ RDIPROGP29 /1:5000
pRabbit/ Osterix/ purified /Santa Cruz /SC-22536R/ 1:200
Mouse/ IgG1 /Purified /Invitrogen /MA1-34581/ 1:200
pGoat/ Mouse /AF633 /Invitrogen /A-21235 /1:200
pRabbit/ Guinea pig /TRITC /OriGene Technologies /R1322T /1:5000
pDonkey/ rabbit /Dylight 550 /Thermofisher /SA5-10039 /1:1000

Validation

All monoclonal antibodies are validated for use in flow cytometry and cell enrichment as well.

Validation	All goat, guinea pig, rabbit and donkey polyclonal antibodies are validated for imaging.<br>Data are available on the manufacturer's websites. The antibodies have been validated by the manufacturers. No additional validation was carried out.

Animals and other research organisms

Policy information about [studies involving animals](); [ARRIVE guidelines]() recommended for reporting animal research, and [Sex and Gender in Research]()

Laboratory animals	Cxcr4+/1013 (+/1013) mice were generated by a knock-in strategy and bred as we described previously. Homozygous Cxcr41013/1013(1013/1013) mice were obtained by crossing heterozygous +/1013 mice. WT mice were used as controls. Adult Boy/J (CD45.1) (Charles River) mice were used as BM donors. Unless specified, all mice were littermates, females and age-matched (8-12 wk-old). All mice were bred in our animal facility under a 12h light/dark cycle, specific pathogen-free conditions (EOPS status) and fed ad libitum. For breeding, mice were in conventional cages with filter top. For experimentation, mice were housed in individually ventilated cages.
Wild animals	No wild animals were used in this study.
Reporting on sex	Unless specified, all mice were littermates, females and age-matched (8-12 wk-old).
Field-collected samples	No field-collected samples were used in the study.
Ethics oversight	All experiments were performed in accordance with the European Union guide for the care and use of laboratory animals and have been reviewed and approved by institutional review committees (CEEA-26, Animal Care and Use Committee, Villejuif, France and Comité d'Ethique Paris-Nord/N°121, Paris, France).

Note that full information on the approval of the study protocol must also be provided in the manuscript.

Flow Cytometry

Plots

Confirm that:

☒ The axis labels state the marker and fluorochrome used (e.g. CD4-FITC).

☒ The axis scales are clearly visible. Include numbers along axes only for bottom left plot of group (a 'group' is an analysis of identical markers).

☒ All plots are contour plots with outliers or pseudocolor plots.

☒ A numerical value for number of cells or percentage (with statistics) is provided.

Methodology

Sample preparation	Mouse SSCs were obtained from bones after centrifugation of intact femurs, tibias and hips to flush out the BM cells. Flushed long bones were cut into fine pieces before enzymatic digestion with 2.5 U/mL collagenase type I (Thermofisher) for 45 min at 37°C under agitation. Released cells were filtered and washed with PBS, 2% FBS (Fetal Bovine Serum). Cell numbers were standardized as total counts per two legs. Peripheral blood was collected by cardiac puncture. Freshly isolated cells were either immunophenotyped, incubated at 37°C for 60 min in RPMI 20 mM HEPES 0.5% BSA (Euromedex) prior to chemokine receptor internalization studies, or expanded in aMEM medium supplemented with 10% FBS, 1% P/S (penicillin 100 Units/mL, streptomycin 100 Units/mL, Gibco) and 50 μM b-mercaptoethanol (PAN biotech). Mouse and human staining analyses were carried out on an LSRII Fortessa flow cytometer (BD Biosciences) using the antibodies (Abs) described above. A Live/Dead Fixable Aqua Dead Cell Stain Kit (Biolegend) was used. To assess the compartmentalization of CXCR4 and ACKR3, human BMSCs were incubated with saturating concentrations of non-conjugated mouse anti-human CXCR4 or ACKR3 Abs, washed in PBS, fixed and permeabilized using the BD Cytofix/Cytoperm Fixation/Permeabilization Kit (BD Biosciences). BMSCs were subsequently stained with anti-CXCR4 and -ACKR3 conjugated mAbs, or the corresponding isotype control, at 4°C for 30 min and then analyzed by flow cytometry.
Instrument	BD LSR Fortessa Flow Cytometer; BD FACSAria III Cell Sorter
Software	FACS Diva software version 7 (BD) were used for collecting data. FLOWJO v10.7 (BD) and GraphPad Prism v8.0e (GraphPad software Inc.) were used for analyzing flow cytometric data.
Cell population abundance	SSC and OPC represented approximately 0.5 and 0.3% of total cells from the bone fraction.
Gating strategy	Gating for SSCs and OPCs flow cytometry analysis and cell sorting:<br>1. Gate on SSC-A vs FSC-A was set to include total bone cells and to exclude debris.<br>2. Gate on SSC-W vs SSC-H was set to exclude doublets.<br>3. Gate on FSC-A vs E506 fixable viability dye allowed to exclude dead cells (E506+).<br>4. Gate on Ter119 vs CD45 was set to exclude erythrocytes, hematopoeitic cells and to select all bone stromal cells (Ter119-CD45-).<br>5. Gate on FSCA-A vs CD31 was set to exclude endothelial cells (CD31+).<br>6. Gate on CD51 and Sca1 was used to select SSCs (CD51+ Sca1+) and OPCs (CD51+ Sca1-).

☒ Tick this box to confirm that a figure exemplifying the gating strategy is provided in the Supplementary Information.
